# Modeling lot-size with time-dependent demand based on stochastic programming and case study of drug supply in Chile

**DOI:** 10.1371/journal.pone.0212768

**Published:** 2019-03-01

**Authors:** Fernando Rojas, Víctor Leiva, Peter Wanke, Camilo Lillo, Jimena Pascual

**Affiliations:** 1 School of Nutrition and Dietetics/Center of Micro-Bio Innovation, Universidad de Valparaíso, Valparaíso, Chile; 2 Business School, Universidad Adolfo Ibáñez, Santiago, Chile; 3 School of Industrial Engineering, Pontificia Universidad Católica de Valparaíso, Valparaíso, Chile; 4 Faculty of Administration, Accounting and Economics, Universidade Federal de Goiás, Goiânia, Brazil; 5 COPPEAD, Universidade Federal de Rio de Janeiro, Rio de Janeiro, Brazil; 6 Department of Computer Sciences, Universidad de Playa Ancha, Valparaíso, Chile; HEC Montréal, CANADA

## Abstract

The objective of this paper is to propose a lot-sizing methodology for an inventory system that faces time-dependent random demands and that seeks to minimize total cost as a function of order, purchase, holding and shortage costs. A two-stage stochastic programming framework is derived to optimize lot-sizing decisions over a time horizon. To this end, we simulate a demand time-series by using a generalized autoregressive moving average structure. The modeling includes covariates of the demand, which are used as predictors of this. We describe an algorithm that summarizes the methodology and we discuss its computational framework. A case study with unpublished real-world data is presented to illustrate the potential of this methodology. We report that the accuracy of the demand variance estimator improves when a temporal structure is considered, instead of assuming time-independent demand. The methodology is useful in decisions related to inventory logistics management when the demand shows patterns of temporal dependence.

## 1 Introduction and bibliographical review

The use of inventory models is important when managing a logistically efficient organization [1–3]. A typical objective for evaluating an inventory system is to minimize the total cost (TC), which is a function of purchase cost, ordering cost per lot (or setup), inventory holding cost, and shortage cost [4]. The inventory system should establish an economic order quantity (EOQ) or lot size to satisfy demand [5, 6]. The EOQ model often considers a single period of decision and assumes a constant rate of demand per unit time (DPUT). Note that this model can also consider multiple periods with more than one level for the decision stages, but in that case the DPUT rate is frequently non-constant. However, the multi-period EOQ model may conduct to an inventory cost greater than that obtained with single-period EOQ model [4].

[7] proposed a framework with dynamic DPUT to decide the optimal order quantity in multiple periods, which is known as the economic lot-sizing (ELS) model, and it is frequently studied in operational research. The ELS model works similarly to the EOQ model, but in a dynamic (and deterministic) setting, that is, over a multi-period planning horizon with non-constant DPUT [6].

Both EOQ and ELS models were conceived assuming deterministic frameworks. However, to formulate more realistic models, the use of a stochastic framework for the DPUT, and its implications for inventory planning, is needed [8]. Under stochasticity, DPUT is considered as a random variable with an associated probability (or statistical) distribution. We refer to this case as the probabilistic (stochastic) ELS inventory model. [9] introduced a probabilistic ELS model using the concept of costs with back-orders. Probabilistic ELS models with and without backlogging were proposed by [10] and [11], respectively. Inventory shortages induced by DPUT uncertainty in ELS models was treated by [12].

The optimization of probabilistic ELS models considers some stochastic elements related to the DPUT [4]. Then, stochastic programming (SP) can be used to solve the optimization problem associated with this probabilistic inventory model [13]. Particularly, two-stage optimization based on SP has been employed in probabilistic ELS and supply chain models; see details in [14] and [15]. More details on two-stage optimization based on SP are provided in Sections 3.1-3.2. [15] considered a two-stage SP and established service level and fill-rate constraints in its second stage, as well as allowed for budget constraints, an aspect often taken into account by the organizations. [16, 17], and [18] utilized two-stage SP to solve non-capacitated (with no budget constraints) ELS models. An evolution of different formulations of the ELS problem is summarized in Table 1, which can be complemented with the recent review provided by [19]. All the articles mentioned in this table assume DPUT to be a time-independent random variable. To the best of our knowledge, there are no papers that consider dependence structures over time for the DPUT in ELS models. We propose a way to find lot-sizes (order quantities) with time-dependent random DPUT based on the work by [15].

**Table 1:** Evolution of literature on ELS formulations and components^⋆^ considered in the mentioned reference.

Reference	Shortage or backorder cost	Uncertain demand	Uncertain lead-time	Decision variable	Constraints	Methodological approach
[5]	No	No	No	ELS	No	Differential calculus
[7]	Yes	Yes	No	ELS over time	No	Heuristic
[9]	Yes	Yes	No	ELS over time	No	Mixed integer linear programming
[20]	No	No	No	ELS, reorder point	No	Linear programming for MRP
[10]	No	No	No	ELS over time	No	Mixed integer dynamic programming
[12]	Yes	Yes	No	ELS over time	Service level with static uncertainty	Mixed integer dynamic programming
[11]	Yes	Yes	No	Plant allocation, ELS	Backlogging	Mixed integer programming with feasibility cut
[21]	No	Yes	No	ELS, safety stock	No	Linear programming for MRP
[22]	Yes	Yes	No	Run order, storage, ELS over time	Service level	One-stage mixed integer programming
[15]	Yes	Yes	No	Run order, ELS, storage and shortage over time	Shortest path, budget	Two-stage robust dynamic SP
[23]	No	Yes	No	ELS over time	Budget	Heuristic with polynomial time
[16]	No	Yes	No	ELS over time	Incremental quantity discount	Multi-stage SP
[24]	Yes	Yes	No	ELS over time	Service level, penalty cost	One-stage dynamic programming
[25]	No	Yes	Yes	ELS, replenishment time	Service level	Linear programming for MRP
[17]	Yes	Yes	Yes	Run order, ELS, storage, shortage	Shortest path with stochastic cost over time	Two-stage mixed integer SP
[26]	No	Yes	No	ELS of product returns, remanufacturing	Inventory balance, return shortest path TCs	Heuristic
[27]	Yes	Yes	No	ELS over time	Service level	Mixed dynamic programming with linear relax
[28]	Yes	Yes	No	Run order, ELS, storage, shortage over time	Two budget constraint with fill-rate	Linear, non-linear SP
[18]	Yes	Yes	Yes	Run order, ELS, storage and shortage over time	Shortest path with acyclic graph	Two-stage SP

^⋆^ order and holding costs were considered in all references and objective function optimized in terms of TC. MRP: material requirements planning.

Since often DPUTs are time-dependent random variables [29], to describe the DPUT of the ELS model adequately, the modeling needs to consider the possible temporal dependence related to the multiple periods and levels of the decision stages for the inventory. This dependence can be modeled by autoregressive moving average (ARMA) time-series. ARMA models are widely flexible, easy to estimate and interpret, and their prediction is straightforward [30]. However, standard ARMA models have a linear structure and a normal (or Gaussian) distribution assumption for the model error. Therefore, standard ARMA models are highly restrictive. When non-normality is detected in the data under analysis, transformations for obtaining normality are frequently employed. Nevertheless, data transformation adds a problem for the interpretation of results. In regression models, this problem was solved in a more general framework of statistical modeling by [31] using generalized linear models (GLM). GLM are based on distributions of the exponential family, of which the normal model is a particular case. GLM do not assume a distribution for the model error, but for the response, and they allow for non-linear structures through a link function that relates the predictor to the model mean. [32] proposed a GLM version of ARMA models known as generalized ARMA (GARMA), which considers ARMA components by transforming the data mean through a link function as in GLM. Then, the past data can be used to improve the accuracy of different models in which statistical aspects are taken into account. In GARMA models, their systematic component allows us to formulate a function of the mean (the link function) by an additive structure of parametric functions, which include explanatory variables (covariates hereafter) and ARMA components. This gives more flexibility to the model formulation, providing the possibility of employing other types of non-linear associations, in addition to the corresponding ARMA framework. GARMA model parameters can be estimated via the maximum likelihood method, once the underlying distribution has been assumed. Often a normal distribution is considered in the modeling of DPUT [33], but other distributions might also be assumed; see, for example, [34]. ARMA and GARMA models are often used to predict future values [29, 32]. GARMA models may also be employed to estimate mean values and find the conditional probability density function to past data, such as it occurs with the DPUT when temporal dependence and covariates are present. This last aspect is of particular interest in probabilistic inventory models.

As DPUT can present temporal dependence, there exists a need to propose inventory models involving this dependence [30, 35]. Such a temporal structure may be added into the inventory costs as part of the objective function or into its constraints. Due to the stochastic nature of the serial dependence of the DPUT values to be modeled (in this case the conditional DPUT over time), SP must be employed in the optimization. Often a method named sample average approximation replaces the original SP problem with Monte Carlo sampling-based methods. Then, the objective function is constructed using scenarios that can be estimated with Monte Carlo sampling. This method assumes that the approximating problem is solved exactly [36]. The optimization may be carried out considering different scenarios for the DPUT, allowing us to generate simulated values (possible realizations or observations) of DPUT with these scenarios. Thus, the simulated values may be clustered with some distance measure [37]. When compared to the standard ELS model, using a two-stage SP approach with time-dependent DPUT can give more flexibility to adjust production or purchase quantities, as well as providing robustness against demand fluctuations [34, 38].

The main objective of this paper is to propose a lot-sizing methodology for an inventory system that faces time-dependent random demands and that seeks to minimize TC as a function of holding and shortage costs. The methodology uses several scenarios based on DPUT data with temporal dependence described by GARMA models. These scenarios consider simulated data which allow the inventory TC to be optimized with a two-stage SP approach. Note that, since the sample average approximation method requires Monte Carlo sampling for generating scenarios, it is necessary to have a random number generator for the GLM and GARMA structures. In the GLM case, there is a method available for this generation. However, for GARMA models, we need to develop a method to generate random numbers that follow this model, which is used only to simulate the data for the experiments. To the best of our knowledge, such a method does not exist in the literature. Thus, a secondary objective of this work is to derive this method.

The paper is organized as follows. Sections 2 and 3 introduce the methodologies which, when combined, allow us to achieve the main objective of the work. Section 2 finishes giving a novel generator of GARMA random numbers covering thus our secondary objective. In Section 4, we provide an algorithm which summarizes the proposed methodology and we describe the computational framework. Then, the numerical results of this article are presented in Section 5. First, a Monte Carlo simulation study is performed to compare the methodology when temporal dependence is present or not in the modeling of DPUT. Second, a case study with unpublished real-world data related to drugs supply in a Chilean hospital is conducted to illustrate the proposed methodology and to show its potential. In Section 6, conclusions on the results obtained in this study, as well as their limitations and future research, are discussed.

## 2 Statistical methodology

In this section, we introduce the statistical methodology utilized here to represent time-dependent random demands in the probabilistic ELS problem. Specifically, we discuss GLM and GARMA structures and then a novel result on generating random numbers that follow a GARMA model is presented. These results have been implemented computationally in a programming language of the R software; see details of this software in Subsection 4.2.

### 2.1 GLM framework

Let *Y* be a random variable related to the DPUT. In addition, consider that the distribution of *Y* belongs to the exponential family of statistical distributions, that is, its probability density function is expressed as

$$f_{Y}\left(y;\vartheta ,\varphi \right)=\text{exp}((y\vartheta -b(\vartheta ))/\varphi +c(y,\varphi )),\;y\in R_{Y},$$ (1)

where *ϑ*, *φ* are canonical and scale parameters, respectively, *R*_*Y*_ is the support of *Y* and *b*, *c* are specific functions defining a particular member of the exponential family. Note that the mean and variance of *Y* can be expressed using first and second derivatives of the function *b*, as well as employing canonical and scale parameters, by E(*Y*) = *b*′(*ϑ*) and Var(*Y*) = *φb*″(*ϑ*), respectively. In GLM, the mean of *Y* is described by a systemic component associated with the link function *g*(*μ*) = *η* and with the values of *r* covariates ***x*** = (*x*_0_, *x*_1_, …, *x*_*r*_)^⊤^, with *x*_0_ = 1, where

$$\mu =E\left(Y\right)=g^{-1}\left(\eta \right)=g^{-1}\left(x^{⊤}\beta \right),$$ (2)

with ***β*** = (*β*_0_, *β*_1_, …, *β*_*r*_)^⊤^ being the regression coefficients associated with ***x***. Now, let *Y*_*t*_ be the random variable of interest *Y* indexed over time *t*, with *t* = 1, …, *n*. Furthermore, consider the conditional distribution of *Y*_*t*_ given the past data set

$$H_{t}=\left\{x_{1},\ldots ,x_{t},y_{1},\ldots ,y_{t-1}\right\},$$ (3)

which is assumed to belong to the exponential family, so that the conditional probability density function according to Eq (1) is expressed as

$$f_{Y_{t}|H_{t}}\left(y_{t};\vartheta_{t},\varphi \right)=\text{exp}(\left(y_{t}\vartheta_{t}-b\left(\vartheta_{t}\right)\right)/\varphi +c\left(y_{t},\varphi \right)),\;y_{t}\in R_{Y_{t}},$$

with the canonical parameter *ϑ*_*t*_ and values of covariates ***x***_*t*_ depending over time *t*, where the parameter *φ* is independent of *t*. We denote the conditional mean and variance of *Y*_*t*_ given ***H***_*t*_ by *μ*_*t*_ = E(*Y*_*t*_|***H***_*t*_) = *b*′(*ϑ*_*t*_) and Var(*Y*_*t*_|*H*_*t*_) = *φb*″(*ϑ*_*t*_), respectively, for *t* = 1, …, *n*.

### 2.2 GARMA models

The GLM framework, connected to the past data set *H*_*t*_ defined in Eq (3), can be formulated in terms of a GARMA model of *p* and *q* orders, denoted by GARMA(*p*, *q*), as

$$g\left(\mu_{t}\right)=\eta_{t}=x_{t}^{⊤}\beta +\sum_{h=1}^{p}\phi_{h}\left(g\left(y_{t-h}\right)-x_{t-h}^{⊤}\beta \right)+\sum_{j=1}^{q}\lambda_{j}\left(g\left(y_{t-j}\right)-\eta_{t-j}\right),$$ (4)

where *ϕ*_*h*_ and λ_*j*_ correspond to the *h*-th and *j*-th components of an ARMA(*p*, *q*) model, related to the autoregressive and moving average components, respectively, and *β* is given as in Eq (2) but now associated with the values of *r* covariates depending over time, denoted by ***x***_*t*_ = (*x*_0_, *x*_1*t*_, …, *x*_*rt*_)^⊤^, with *x*_0_ = 1. The link function *g*(*μ*_*t*_) = *η*_*t*_ of the GARMA model given in Eq (5) can be, for example, the identity function (to represent linear association), the inverse function or the logarithmic –log– function (to represent non-linear association), whereas the corresponding model variance is assumed to be constant over time. In the case of the identity link function, we have that

$$\begin{array}{c} \mu_{t}=x_{t}^{⊤}\beta +\sum_{h=1}^{p}\phi_{j}\left(y_{t-h}-x_{t-h}^{⊤}\beta \right)+\sum_{j=1}^{q}\lambda_{j}\left(y_{t-j}-\mu_{t-j}\right). \end{array}$$ (5)

Now, consider the martingale residual [32], *υ*_*t*_ = *y*_*t*_ − *μ*_*t*_, which are uncorrelated and have marginal mean equal to zero. By expressing $w_{t}=y_{t}-x_{t}^{⊤}\beta$ and based on Eq (5), we have

$$w_{t}=\sum_{h=1}^{p}\phi_{j}w_{t-h}+\sum_{j=1}^{q}\lambda_{j}\upsilon_{t-j}+\upsilon_{t}.$$ (6)

Considering lag operators (*L*) corresponding to the autoregressive and moving average components given by

$$\begin{array}{ccc} \Phi (L) & = & 1-\phi_{1}L^{1}-\phi_{2}L^{2}-\cdots -\phi_{p}L^{p},\\ \Lambda (L) & = & 1+\lambda_{1}L^{1}+\lambda_{2}L^{2}+\cdots +\lambda_{q}L^{q}, \end{array}$$

respectively, we may rewrite Eq (6) as

$$w_{t}=\frac{\Lambda (L)}{\Phi (L)}\upsilon_{t}=\Psi \left(L\right)\upsilon_{t},$$

where Ψ(*L*) = Λ(*L*)/Φ(*L*) = 1 + *ψ*_1_*L*^1^ + *ψ*_2_*L*^2^ + ⋯, assuming that Φ(*L*) is invertible. In this context, [32] demonstrated that the corresponding marginal mean and variance, E(*Y*_*t*_) and Var(*Y*_*t*_) namely, are defined by

$$\begin{array}{ccc} E(Y_{t}) & = & E\left(x_{t}^{⊤}\beta +w_{t}\right)=E\left(x_{t}^{⊤}\beta \right)+E\left(w_{t}\right)=x_{t}^{⊤}\beta ,\\ \text{Var}(Y_{t}) & = & \text{Var}\left(w_{t}\right)=E\left(w_{t}^{2}\right)=\varphi E\left(\Psi^{\left(2\right)}\left(L\right)\text{Var}\left(Y_{t}|H_{t}\right)\right), \end{array}$$ (7)

where $\Psi^{\left(2\right)}\left(L\right)=1+\psi_{1}^{2}L^{1}+\psi_{2}^{2}L^{2}+\cdots ,$ for all stationary time-series.

**Remark 1** *From* Eq (7), *note that the marginal mean E*(*Y*_*t*_ *) does no depend on past data*. *However, since the term* Ψ^(2)^(*L*) ≥ 1, *then Var*(*Y*_*t*_) ≥ *Var*(*Y*_*t*_|***H***_*t*_), *that is, the conditional variance on past data is less than or equal to the marginal variance, which does not consider the temporal disposition of the observations. This is fundamental to improve the accuracy of the conditional variance based on past data* [32].

Given *n* observations *y*_1_, …, *y*_*n*_ of *Y*_*t*_, for *t* = 1, …, *n*, the corresponding likelihood function is constructed as the product of conditional probability density functions of *Y*_*t*_ given the past data ***H***_*t*_. Thus, if ***θ*** = (***β***^⊤^, *ϑ*_*t*_, *φ*, *ϕ*^⊤^, **λ**^⊤^)^⊤^ is the vector of model parameters to be estimated, the associated log-likelihood function for ***θ*** is given by

$$\begin{array}{c} \ell \left(\theta \right)=\sum_{t=1}^{n}\text{log}(f_{Y_{t}|H_{t}}\left(y_{t};\theta \right)). \end{array}$$ (8)

To obtain the maximum likelihood estimate $\hat{\theta }$ of ***θ***, we must take derivatives of Eq (8) with respect to each parameter ***β***, ***ϑ***_*t*_, ***φ***, ***ϕ*** and **λ**. Inference (confidence intervals and hypothesis testing) about ***θ*** can be based on the asymptotic normality of the maximum likelihood estimator $\hat{\theta }$.

### 2.3 Model checking and diagnostic

The quantile residual is often used in GARMA models as a diagnostic tool to detect their adequacy to the data, which is defined as

$$r_{t}=\Phi^{-1}\left(F_{Y_{t}|H_{t}}\left(y_{t};\hat{\theta }\right)\right),$$ (9)

where $F_{Y_{t}|H_{t}}$ is the cumulative distribution function of *Y*_*t*_ conditional to past data, $\hat{\theta }$ is the maximum likelihood estimate of ***θ***, and Φ^−1^ is the inverse cumulative distribution function of a standard normal distribution. Note that the quantile residual follows the standard normal distribution. For more details about this residual, see [39].

GARMA models have three elements: (i) the distribution of the response; (ii) the link function for the mean; and (iii) a linear predictor containing a set of model parameters, corresponding to regression coefficients associated with the covariates, as well as autoregressive and moving average coefficients. For a specific data set, the building process of a GARMA model consists of comparing several competing models based on different combinations of these elements. The deviance is used as an indicator to compare these models, which is minus two times the log-likelihood ratio of the reduced model (in our case GLM) and the full model (in our case the GARMA model). We can employ model selection tools, such as Akaike (AIC) and Bayesian (BIC) information criteria, to select the best GARMA model. AIC and BIC allow us to compare models by the expressions

$$\text{AIC}=-2\ell \left(\hat{\theta }\right)+2m,\;\text{BIC}=-2\ell \left(\hat{\theta }\right)+m\;\text{log}\left(n\right),$$

with $\ell (\hat{\theta })$ being the log-likelihood function evaluated at $\theta =\hat{\theta }$ and *n*, *m* being the sample size and number of model parameters, respectively. AIC and BIC correspond to a penalized log-likelihood function as the model has more parameters, making it more complex. A smaller AIC or BIC indicates a better model; for more details about deviance, AIC and BIC, see [40].

### 2.4 Generator of GARMA random numbers

Algorithm 1 introduces a novel generator of random numbers from a GARMA model. Note that random numbers following a GLM can be obtained by fixing the orders *p* = 0 and *q* = 0 in the GARMA model, that is, GLM ≡ GARMA(0, 0) model.

**Algorithm 1** Random number generator from a GARMA model

1: Construct the GLM term of the GARMA model by fixing:

 1.1 A number *r* of covariates and the size sample *n*.

 1.2 Values for the regression coefficients ***β*** associated with the *r* covariates.

 1.3 The link function *g*.

2: Build the autoregressive term of the GARMA model by fixing:

 2.1 The dimension *p* of the autoregressive parameters *ϕ*, assuming *p* = 0 if a moving average model is of interest.

 2.2 Values for autoregressive coefficients *ϕ*.

3: Establish the moving average term of the GARMA model by fixing:

 3.1 The dimension *q* of the moving average parameters **λ**, assuming *q* = 0 if an autoregressive model is of interest.

 3.2 Values for moving average coefficients **λ**.

4: Assume a distribution for *Y*_*t*_ within the exponential family and fix its parameter *φ* (note that by fixing *φ* we choose a specific member of the exponential family and then formulate the GARMA model).

5: Obtain values for the covariate matrix ***X*** = (*x*_*tk*_), where each of its *k* columns ***x***_⋅*k*_ = (1, *x*_1*k*_, …, *x*_*nk*_)^⊤^ is randomly generated from the uniform distribution in [0, 1] using the Monte Carlo method.

6: Also using the Monte Carlo method, generate a time-series from a GARMA model with *t* = 1, …, *n* following the steps:

 6.1 For *t* = 1, generate a value of *y*_*t*_ from $Y_{t}|H_{t}∼F\left(\mu_{t},\varphi \right)$, where F is a member of the exponential family of parameter *φ* (as defined in Step 4) and $\mu_{t}=g^{-1}\left(x_{t}^{⊤}\beta \right)$, with *g* and *β* defined in Step 1 and ***x***_*t*_ in Eq (3) but numerically calculated from Step 5. Note that definition of *μ*_*t*_ is similar to the expression given in Eq (5) for *p* = 0 and *q* = 0 (with the identity link function).

 6.2 For each *t* from 2 to *n*:

  6.2.1 Obtain the autorregresive term using

$$\begin{array}{c} a_{t}=\{\begin{array}{c} \sum_{h=1}^{p}\phi_{h}u_{h},\;\;p>0;\\ 0,\;\;p=0; \end{array}\;\text{with}\;u_{h}=\{\begin{array}{c} g\left(y_{t-h}\right)-x_{t-h}^{⊤}\beta ,\;\;t>h;\\ 0,\;\;t\leq h. \end{array} \end{array}$$

  6.2.2 Calculate the moving average term employing

$$\begin{array}{c} b_{t}=\{\begin{array}{c} \sum_{j=1}^{q}\lambda_{j}v_{j},\;\;q>0;\\ 0,\;\;q=0; \end{array}\;\text{with}\;v_{j}=\{\begin{array}{c} g\left(y_{t-j}\right)-\eta_{t-j},\;\;t>j;\\ 0,\;\;t\leq j. \end{array} \end{array}$$

  6.2.3 Compute *μ*_*t*_ = *g*^−1^(*η*_*t*_) as location parameter indexed over time *t*, with $\eta_{t}=x_{t}^{⊤}\beta +a_{t}+b_{t},$ using *a*_*t*_ and *b*_*t*_ defined in Steps 6.2.1-6.2.2, respectively, and *η*_*t*_ being analogous to expression given in Eq (4).

  6.2.4 Generate a value of *y*_*t*_ similarly as in Step 6.1, but now *μ*_*t*_ is computed as in Step 6.2.3.

## 3 Stochastic programming methodology

In this section, we present the SP methodology to find the ELS in *T* periods of decision stages assuming a time-dependent random DPUT. This methodology has been implemented in the R software.

### 3.1 Stochastic programming formulation

Table 2 summarizes the elements of the two-stage probabilistic ELS model. In this table, both *Z*_*t*_ and *Q*_*t*_ are considered as first stage variables, while the variables *I*_*t*_ and *S*_*t*_ are considered as second stage variables. In the first stage, we decide whether or not to purchase and how much to purchase, whereas in the second stage, after observing the demand, we obtain inventory and shortage levels. The corresponding SP framework used to minimize the expected TC –E(TC)– of the inventory model can be formulated as [15]

$$\text{min}\left\{E\left(\text{TC}\right)\right\}=\text{min}\{\sum_{\omega \in \Omega }\sum_{t=1}^{T}p_{t}^{\omega }\left(o_{t}Z_{t}+u_{t}Q_{t}+h_{t}I_{t}^{\omega }+s_{t}S_{t}^{\omega }\right)\},$$ (10)

subject to

$$\begin{array}{ccc} Q_{t}+\left(I_{t-1}^{\omega }-S_{t-1}^{\omega }\right)-\left(I_{t}^{\omega }-S_{t}^{\omega }\right) & = & y_{t}^{\omega },\\ Q_{t} & \leq & C_{t}Z_{t}, \end{array}$$ (11)

$$\begin{array}{c} \forall t\in T,\;\;\forall \omega \in \Omega ,\;\;Q_{t}\geq 0,\;\;I_{t}^{\omega }\geq 0,\;\;S_{t}^{\omega }\geq 0,\;y_{t}^{\omega }\geq 0,\;\;p_{t}^{\omega }\in \left[0,\;1\right],\;\;Z_{t}\in \left\{0,\;1\right\}, \end{array}$$

where Ω is the set of selected possible demand scenarios and *ω* is a specific scenario, with a fixed number of scenarios in each period of the decision stages. The objective function defined in Eq (10) attains a solution that minimizes E(TC) over all scenarios. This minimization can be carried out through the addition of sharing cuts for feasibility and optimality at the resource function, whenever this function or its constraints contain stochastic coefficients that exhibit inter-stage dependency of ARMA type in a multi-stage problem [41]. The approach defined in Eq (10) can be reformulated using the L-shaped method, according to [42], as a master problem in each period *t* of the decision variables in two stages by means of

$$\text{min}\left\{o_{t}Z_{t}+u_{t}Q_{t}+\gamma_{2}\left(Z_{t},Q_{t}\right)\right\},\;\gamma_{2}\in R,$$ (12)

subject to *u*_*t*_ *Q*_*t*_ ≤ *Z*_*t*_ *C*_*t*_, where the decision variables of the first stage *Z*_*t*_ and *Q*_*t*_ are fixed momentarily until the problem of second stage is solved. Note that

$$\gamma_{2}\left(Z_{t},Q_{t}\right)=\text{min}\left\{p_{t}^{\omega }\left(h_{t}I_{t}^{\omega }+s_{t}S_{t}^{\omega }\right)\right\}$$ (13)

defined in Eq (12) represents the objective function of the second stage (considered here as a subproblem), which is a function in each period *t* of the decision variables in the first stage, subject to

$$Q_{t}+\left(I_{t-1}^{\omega }-S_{t-1}^{\omega }\right)-\left(I_{t}^{\omega }-S_{t}^{\omega }\right)=y_{t}^{\omega },\;\forall \omega \in \Omega .$$ (14)

The subproblem defined in Eq (13), with its constrains given in Eq (14), have a dual form

$$\gamma_{2}\left(Z_{t},Q_{t}\right)=\text{max}\left\{\pi_{1}\left(0-0\times Z_{t}\right)+\pi_{2}\left(y_{t}^{\omega }-Q_{t}\right)\right\},$$ (15)

subject to *π*_1_ ≤ *h*_*t*_ and *π*_2_ ≤ *s*_*t*_, where *π*_1_ and *π*_2_ are the dual variables related to these constrains. Next, we indicate how the cuts are generated and added to solve the problem using the L-shaped method. It is known that the optimal solution of a linear programming, if it exists, is attained at a vertex. Let $\Theta_{1}=\left(\pi_{1}^{\left(1\right)},\ldots ,\pi_{1}^{\left(v\right)}\right)$ and $\Theta_{2}=\left(\pi_{2}^{\left(1\right)},\ldots ,\pi_{2}^{\left(v\right)}\right)$ be a finite set of vertices related to *π*_1_ and *π*_2_, respectively. Therefore, the master and dual problems defined in Eqs (12) and (15), respectively, can be solved by *γ*_2_(*Z*_*t*_, *Q*_*t*_) = min{*γ*_2_}, subject to

$$\begin{array}{ccc} \gamma_{2}\geq \pi_{1}^{\left(1\right)}\left(0-0\times Z_{t}\right) & + & \pi_{2}^{\left(1\right)}\left(y_{t}^{\omega }-Q_{t}\right),\\ & \vdots & \\ \gamma_{2}\geq \pi_{1}^{\left(1\right)}\left(0-0\times Z_{t}\right) & + & \pi_{2}^{\left(v\right)}\left(y_{t}^{\omega }-Q_{t}\right), \end{array}$$

where the constraints are called cut tangent planes to the objective function at each point (*Z*_*t*_, *Q*_*t*_). This is a convex external approximation of the resource function, with *γ*_2_ varying freely. It is possible to solve this mixed integer linear programming defined in Eq (12), often written in short as MILP, by using the lpSolveAPI package of the R software.

**Table 2:** Elements of the ELS model.

Parameters	Variables
*t*: Period index of the decision stage in the planning time horizon (*t* = 1, …, *T*).	*Z*_*t*_: Binary variable indicating whether a purchase is carried out in period *t* or not.
*C*_*t*_: Purchase budget in period *t*.	*Q*_*t*_: Quantity of units to be purchased in period *t*.
*u*_*t*_: Unitary cost of purchase in period *t*.	*I*_*t*_: Stock level at the end of period *t*.
*o*_*t*_: Fixed order cost in period *t*.	*I*_0_: Initial stock level.
*h*_*t*_: Holding cost at the end of period *t*.	*S*_*t*_: Shortage level at the end of period *t*.
*s*_*t*_: Shortage cost at the end of period *t*.	
$p_{t}^{\omega }$: Probability of occurrence of the scenario *ω* in period *t* of the decision stage.	

### 3.2 Scenarios in two stages

One way of introducing randomness in SP problems is by generating a finite number of scenarios as follows. First, a distribution with estimated or known parameters is assumed, which approximates the true distribution of the DPUT. Second, a large number *N* of values for the DPUT (for example, *N* = 10000) is simulated. Third, a number *S* of scenarios must be established to represent the inherent variability of the DPUT (for example, *S* = 100). A specification of *S* is reached by clustering *N* values into *S* nodes, which correspond to the centroids of each cluster and are represented in a diagram with the associated probabilities over the nodes [37]. SP provides good solutions if the assumed distribution for the DPUT is adequate statistically.

Recall that, in the two-stage SP model, each level of a scenario tree is a decision stage. Consequently, a two-stage SP model corresponds to a scenario tree with only two levels in each time period. In the first stage of this model, as mentioned, the decision variables are whether to purchase or not, represented by the binary variable *Z*_*t*_, and which ELS must be purchased, represented by the variable *Q*_*t*_, both of which are decided before knowing the values of DPUT *Y*_*t*_. In the second stage, the decision variables are the inventory level *I*_*t*_ and the shortage level *S*_*t*_, which are generated after knowing *Y*_*t*_. The representation of each scenario *ω* ∈ Ω is constructed as follows. In each period *t*, the primary node shows the first decision stage and the secondary nodes display the second decision stage with the values of *Y*_*t*_ and their respective probabilities $p_{t}^{\omega }$ indicated over the nodes [43].

To obtain the values presented in each node and time *t*, as well as their respective probabilities, we utilize the values ${\hat{Y}}_{t}$ fitted from GLM or GARMA structures as seeds to simulate 1000 values of *Y*_*t*_. Here, GLM and GARMA parameters required for the simulation can be estimated by the maximum likelihood method using real-world data. These estimates are considered as constant over *t*. Then, the 1000 simulated values in each period *t* of the second decision stage are grouped employing non-hierarchical clustering. The analysis of non-hierarchical groups aims to find a clustering of objects. In our case, the objects are values of simulated demands, such that the separation of these groups or clusters is maximized while minimizing the distances within the group in relation to its average or centroid. We use the *k*-means method to assign simulated demands to a number of groups defined by the user (for example *k* = 100), which form the scenarios for the SP [44]. We occupy the Ward method [45] to group such that the Euclidean distances between the simulated elements to be clustered is minimized. For more details of non-hierarchical grouping, see [44]. In our case, the possible values to be considered in each node are the centroids of this non-hierarchical clustering, while the probabilities of each branch correspond to the proportion of grouped elements in each centroid with respect to the total of simulated values. Note that the simulated values from a GLM and GARMA structures can be obtained with Algorithm 1 presented in Section 2.

To reach a range of solutions from the SP proposed in Eqs (10) and (11), we apply the approach described above to cases of extreme demand percentiles. Note that considering this is a purely statistical issue, because the 5-th and 95-th percentiles can be used as extreme values of the distribution. In statistics, any value below 5% is considered small and above 95% is large. Thus, we intend to demonstrate that a more accurate range of variables in the first (*Q*_*t*_) and second (*I*_*t*_, *S*_*t*_) decisions, as well as E(TC), are obtained when comparing results of the SP proposed in Eqs (10) and (11) to describe the DPUT with GARMA vs. GLM. Specifically, we make this comparison by obtaining the solutions of Eqs (10) and (11), considering 5-th and 95-th percentiles of the distribution of *Y*_*t*_ (described by GARMA and GLM), for *t* = 1, 2, 3. We denote the *l*-th percentile of the distribution of *Y*_*t*_ by $y_{t_{l\times 100}}^{\omega }$ for the scenario *ω* in period *t* of the second decision stage. Then, $y_{t_{5}}^{\omega }$ and $y_{t_{95}}^{\omega }$ are the 5-th and 95-th percentiles, respectively, each in their respective scenario *ω* of the second decision stage in period *t*. Given that the distribution parameters are known, we can simulate values of these percentiles in each period *t*, clustering the data to obtain their value and probability in each node of the second stage; see details of this procedure in steps 3 and 4 of Algorithm 2, and of the generation of SP scenarios in [37].

### 3.3 Evaluation of SP by using out-of-sample scenarios

To evaluate the purchase plan of the ELS obtained from SP models based on generation of scenarios, it is possible to apply the optimization to a realistic framework of the rolling horizon using out-of-sample scenarios. To generate these scenarios, we use the same distribution and parameters considered in the scenario representation mentioned in Section 3.2. Note that the objective function of the SP does not provide the true cost to be incurred when implementing the solution in a real environment, with out-of-sample data within a rolling horizon framework. Then, we can contrast the value of a solution in stochastic scenarios with respect to a solution obtained in a deterministic scenario, comparing the percentage increase of the TC we pay for ignoring uncertainty [46]. This cost is computed as (DC − SC)/SC, where DC and SC are the TCs accumulated over each simulation run for the deterministic and stochastic models, respectively. Repeating this comparative experience in *J* instants, we obtain the average percentage cost increase of the deterministic and stochastic solutions (Δ) and its mean absolute deviation (MAD) as

$$\Delta =\frac{1}{J}\sum_{j=1}^{J}(\frac{{\text{DC}}_{j}-{\text{SC}}_{j}}{{\text{SC}}_{j}})\times 100\%,\;\text{MAD}=\frac{1}{J}\sum_{j=1}^{J}|\frac{{\text{DC}}_{j}-{\text{SC}}_{j}}{{\text{SC}}_{j}}|\times 100\%.$$

## 4 Summary of methodological and computational aspects

In this section, we condense, in an algorithm, the proposed methodology constructed from Sections 2 and 3. Then, the computational framework used to implement this methodology is described.

### 4.1 Summary of the optimization methodology

To obtain the observed values $y_{t}^{\omega }$ of *Y*_*t*_, their probabilities $p_{t}^{\omega }$, and E(TC), we summarize in Algorithm 2 the methodology used to get the inventory TC over *T* periods of the decision stages.

**Algorithm 2** Summary of the methodology to optimize TC over *T* periods of decision stages

1: Collect data *y*_1_, …, *y*_*n*_ of the response *Y* and data *x*_1*j*_, …, *x*_*nj*_ of the covariate *X*_*j*_, with *j* = 1, …, *r*.

2: Perform an exploratory data analysis according to the following steps:

  2.1 Compute descriptive statistics for *y*_1_, …, *y*_*n*_ to identify the type of data distribution. If evidence of symmetry exists, use a normal distribution. Else, employ an asymmetric distribution such as the gamma model. In both cases, continue with Step 2.2.

 2.2 Detect autocorrelation for *y*_1_, …, *y*_*n*_ using the autocorrelation (ACF) and partial autocorrelation (PACF) functions. If evidence of autocorrelation exists, utilize an ARMA model of adequate orders *p*, *q* and continue with Step 2.3. Else, assume other models.

 2.3 Construct scatter-plots between *Y* and each covariate *X*_*j*_ to obtain the kind of association to be considered in the modeling. If evidence of correlation between *Y* and some covariate *X*_*j*_ exists, employ regression models and continue with step 3. Else, do not consider the covariate(s).

3: Formulate GARMA models and estimate their parameters according to the following steps:

 3.1 Consider a suitable link function for the GARMA model based on step 2.3.

 3.2 Estimate the GARMA parameters (***θ***) using the maximum likelihood method.

 3.3 Select the best GARMA model considering the smallest value for an information criteria (AIC or BIC) and the deviance, producing the fitted model $\hat{y}$ with its corresponding values ${\hat{y}}_{1},\ldots ,{\hat{y}}_{n}$.

 3.4 Obtain the *l* × 100-th percentile, *y*_*l*×100_ namely, of the distribution of *Y* using the estimated parameters ***θ***, which are calculated in step 3.2.

4: Generate cluster scenarios carrying out a “for cycle” from *t* = 1 to *t* = *T* periods of the decision stages following the steps:

 4.1 Fix *l* = *l*_1_ at a small value (for example, *l*_1_ × 100 = 5) and simulate *n* data $y_{1_{l_{1}\times 100}}^{⋆},\ldots ,y_{n_{l_{1}\times 100}}^{⋆}$ of the distribution of *Y* using the Monte Carlo method, with mean *y*_*l*_1_×100_ and estimated parameters $\hat{\theta }$.

 4.2 Conduct a cluster analysis on $y_{1_{l{}_{1}\times 100}}^{⋆},\ldots ,y_{n_{l_{1}\times 100}}^{⋆}$ with a number of 100 scenarios conformed for the clusters and obtain $y_{t}^{\omega }$ and $p_{t}^{\omega }$, for *ω* = 1, …, 2^*t*^, where $y_{t}^{\omega }$ is the *ω*-th cluster centroid (scenario), and $p_{t}^{\omega }$ is the probability of occurrence of this scenario.

 4.3 Fix *l* = *l*_2_ at a large value (for example, *l*_2_ × 100 = 95) and simulate *n* data $y_{1_{l_{2}\times 100}}^{⋆},\ldots ,y_{n_{l_{2}\times 100}}^{⋆}$ of the distribution of *Y* using once again the Monte Carlo method, with mean *y*_(*l*_2_ × 100)_ and estimated parameters $\hat{\theta }$.

 4.4 Conduct a cluster analysis on $y_{1_{l_{2}\times 100}}^{⋆},\ldots ,y_{n_{l_{2}\times 100}}^{⋆}$ with a number of 100 scenarios conformed for the clusters and obtain $y_{t}^{\omega }$ and $p_{t}^{\omega }$, for *ω* = 1, …, 100, where $y_{t}^{\omega }$ is the *ω*-th cluster centroid (scenario), and $p_{t}^{\omega }$ is the probability of occurrence of this scenario.

5: Set values for the components *u*_*t*_, *o*_*t*_, *h*_*t*_ and *s*_*t*_ of the inventory model given in Eq (10) and the components *C*_*t*_ and *I*_0_ of the constrains given in Eq (11).

6: Optimize the model given in Eq (10) to obtain *Z*_*t*_, *Q*_*t*_, $I_{t}^{\omega }$ and $S_{t}^{\omega }$, denoted by ${\tilde{Z}}_{t}$, ${\tilde{Q}}_{t}$, ${\tilde{I}}_{t}^{\omega }$ and ${\tilde{S}}_{t}^{\omega }$, respectively.

7: Establish the optimum inventory TC as $\tilde{E}\left(\text{TC}\right)=\sum_{\omega \in \Omega }\sum_{t=1}^{T}p_{t}^{\omega }\left(o_{t}{\tilde{z}}_{t}+u_{t}{\tilde{Q}}_{t}+h_{t}{\tilde{I}}_{t}^{\omega }+s_{t}{\tilde{S}}_{t}^{\omega }\right)$.

### 4.2 Computational framework

We implement the methodology in the R software, a non-commercial and open source package for statistics and graphs which can be secured from www.r-project.org. The R software is currently very popular in the international scientific community. For an application of R in inventory models; see [47]. Some R packages related to statistical distributions that may be useful in inventory models are available in CRAN.R-project.org; see, for example, [40] and [48]. Specifically, we utilize the base package for descriptive statistics, the gamlss.util package to perform a statistical analysis on DPUT values and time-series analysis for GARMA models. We use the RcmdrMisc package for cluster analysis in the setting of inventory models, and the lpSolveAPI package to solve the linear programming with the L-shaped method based on Eq (10), which can also be solved by using several commercial software packages as LINGO and CPLEX. Note that by occupying 100 scenarios of DPUT in three periods of the second decision stage, the processing time is 30 seconds in a computer with the features detailed in Table 3.

**Table 3:** Characteristics of the computer used in the simulations.

Characteristic	Description
Operating system	Windows 10 home single language 64 bits (10.0, compilation 17134)
Model	80FY, BIOS A7CN44WW
Processor	INTEL (R) Pentium (R) CPU N3540 @ 2.16 GHz (4CPUs)
RAM	8192 MB

## 5 Numerical results

In this section, we first conduct a Monte Carlo simulation study, which allows us to compare the performance of ELS and its inventory TC for different methods when describing DPUT based on GARMA and GLM structures. Second, real-world DPUT data from a drug supply case study in a Chilean public hospital are analyzed with the proposed methodology.

### 5.1 Simulation study

To carry out the aforementioned simulation study, we consider: (i) two samples, one of time-dependent DPUT and time-independent DPUT, both using Algorithm 1, are generated; (ii) a range of SP solutions obtained using extreme demand percentiles (5-th and 95-th) are obtained for both decision variables and TCs; and (iii) inventory TC for the ELS model are evaluated by Algorithm 2. We obtain the TC over *T* = 3 periods of the decision stages and *n* = 100 by employing GARMA and GLM structures for simulated data with temporal dependence, based on normal and gamma distributions, different link functions and diverse values for standard deviations (SDs). We describe the time-dependent DPUT mean considering the GARMA(1, 1) structure given by

$$g\left(\mu_{t}\right)=\eta_{t}=\beta_{0}+\beta_{1}x_{t}+\phi (y_{t-1}-\beta_{0}-\beta_{1}x_{t-1})+\theta (y_{t-1}-\eta_{t-1}),\;t=1,\ldots ,100.$$ (16)

In addition, as comparison for time-independent DPUT, we use the GLM defined as

$$g\left(\mu_{t}\right)=\eta_{t}=\beta_{0}+\beta_{1}x_{t},\;t=1,\ldots ,100.$$ (17)

In both models, we employ link functions: *g* ≡ {identity, log}. As mentioned in Algorithm 2, we assume that the values *x*_*t*_ of the covariate *X*_*t*_ are obtained from a uniform distribution in the interval [0, 1]. The values assumed for *ϕ* and *θ* in Eq (16) are *ϕ* = 0.5 and *θ* = 0.25. The number of Monte Carlo replications are 10000. In each of these replications: (i) we obtain the observations ***y*** = (*y*_1_, …, *y*_100_)^⊤^ for the normal and gamma distributions with temporal dependence by using Algorithm 1; and (ii) we fit GARMA and GLM structures by estimating their parameters and obtaining fitted values of Eqs (16) and (17), respectively. These fitted values are computed for different fixed values of the corresponding distribution SD, *σ* namely, and regression coefficients *β*_0_, *β*_1_. To compare different distributions, we set diverse configurations for GARMA and GLM structures; see Table 4.

**Table 4:** Distributions and true parameters used in the simulation study.

Distribution	Link function	*β*_0_	*β*_1_	*σ*
Normal	Identity	500	2	{5, 10, 15, 20}
Normal	Log	5	1	{5, 10, 15, 20}
Gamma	Identity	500	2	{0.1, 0.25, 0.5, 0.75}
Gamma	Log	5	1	{0.1, 0.25, 0.5, 0.75}

We obtain the 5-th and 95-th percentiles of the DPUT distribution for each period of the decision stages under analysis, by using the quantile function implemented in the gamlss package with qGA and qNO commands for gamma and normal distributions, respectively. For each of the replications, we consider three periods by using three consecutive fitted values as mean of the distributions in each period. The SD is assumed to be constant in each period of the decision stages and estimated by GARMA and GLM structures. Subsequently, by applying Algorithm 2 (from steps 4 to 7): (i) we generate scenarios using simulation with a sample size 1000 in each node; and (ii) we obtain the ELS with its TC over all periods of the decision stages. Fixed values for parameters in all periods of the decision stages are reported in Table 5.

**Table 5:** Inventory model parameters for *t* = 1, 2, 3 periods of the decision stages.

Inventory element	Value
Fixed order cost in period *t*:	*o*_*t*_ = 0.75
Shortage cost at the end of period *t*:	*s*_*t*_ = 0.5
Holding cost at the end of period *t*:	*h*_*t*_ = 0.05
Unitary purchase cost in period *t*:	*u*_*t*_ = 0.5
Purchase budget in period *t*:	*C*_*t*_ = 1500
Percentiles of DPUT:	*l* × 100 = {5, 95}

Through this simulation study, as mentioned, we evaluate $\tilde{E}\left(\text{TC}\right)$ for two DPUT distributions and two link functions. We are interested in describing the empirical distribution of $\tilde{E}\left(\text{TC}\right)$ (for reasons of space, we omit the analysis of the decision variables of first and second stage). The results on the statistical properties of the maximum likelihood estimators ${\hat{\beta }}_{0}$, ${\hat{\beta }}_{1}$, $\hat{\sigma }$ and $\hat{\phi }$ are not provided here, but these properties can be found in the simulation study presented in [32]. Through this study, we intend to corroborate the differences between the results of the $\tilde{E}\left(\text{TC}\right)$ when describing the DPUT with GLM and GARMA structures. On the one hand, we make explicit that the variability of these results, reflected in the range of values of the obtained solutions given the extreme demand percentiles under consideration, should always be smaller in the GARMA model than in the GLM, which would provide more accurate results with our proposal. On the other hand, we compare the medians of $\tilde{E}\left(\text{TC}\right)$ using the Friedman test, with both models for the DPUT and link functions being considered. Table 6 reports the mean, SD, coefficients of skewness (CS), kurtosis (CK), median, and Friedman *p*-value of $\tilde{E}\left(\text{TC}\right)$ for the indicated distribution and link function. Note that, when the parameter *σ* (related to the SD of the DPUT distribution) increases, the range of $\tilde{E}\left(\text{TC}\right)$ obtained from the 5-th and 95-th percentiles is smaller in the GARMA model than in GLM, as indicated in Remark 1. Observe that $\tilde{E}\left(\text{TC}\right)$ under time-independent DPUT is greater than in the case of time-dependent random DPUT, which is explained by higher inventory levels to cover a higher DPUT uncertainty [49]. Then, the conditional DPUT variance obtained from GARMA models under a normal distribution and identity link is smaller than the marginal DPUT variance generated from GLM, which leads to smaller values of $\tilde{E}\left(\text{TC}\right)$. (Similar results are obtained for models with a logarithmic link function.) Notice that $\tilde{E}\left(\text{TC}\right)$ for the case of the gamma distribution presents the largest SD. With respect to the SD, note that, if the DPUT presents temporal dependence, then the SD of the GARMA model is smaller than in GLM, for any distribution and link function considered. However, when a non-identity link function is used, these values are similar. In this simulation study, we find that not only the DPUT variance estimate is smaller when employing GARMA models under temporal dependence, but also the estimator of min{E(TC)} defined in Eq (10) has a smaller variance. Therefore, the best accuracy is obtained when we fit a GARMA model using temporal dependence of the DPUT instead of inventory models obtained under time-independent DPUT.

**Table 6:** Empirical mean, SD, CS, CK, median, and Friedman *p*-value of the $\tilde{E}\left(\text{TC}\right)$ for the indicated distribution, link, *σ* and percentile.

Distribution-link	*σ*	Mean		SD		CS		CK		Median		Friedman
		GARMA	GLM	GARMA	GLM	GARMA	GLM	GARMA	GLM	GARMA	GLM	*p*-value
		*l* × 100 = 5										
Normal-identity	5	744.21	742.85	3.57	3.87	-0.003	-0.21	0.20	-0.35	744.37	742.78	<0.001
	10	736.96	733.57	6.19	7.02	-0.03	0.06	0.28	0.14	735.91	733.33	<0.001
	15	727.39	722.74	11.23	11.76	-0.15	-0.06	0.18	-0.16	727.21	721.78	<0.001
	20	718.44	713.61	12.88	16.21	-0.13	-0.28	0.88	-0.31	717.75	714.17	<0.001
Gamma-identity	0.10	665.29	651.12	27.12	28.44	-0.04	-0.21	-0.04	0.44	663.11	650.42	<0.001
	0.25	542.89	504.18	55.22	60.98	1.12	-0.04	4.12	-0.45	544.12	504.16	<0.001
	0.50	318.44	271.77	83.27	89.20	0.15	0.35	-0.19	-0.65	315.89	268.34	<0.001
	0.75	147.79	127.22	71.01	78.03	0.88	0.28	0.16	-0.32	148.26	126.21	<0.001
Normal-log	5	448.56	441.09	2.97	3.09	-0.70	-0.42	1.12	-0.28	447.32	442.32	<0.001
	10	562.12	555.21	11.34	12.22	0.19	0.22	0.39	-0.05	561.99	554.23	<0.001
	15	708.02	693.71	17.66	19.02	-0.30	-0.67	-0.14	0.92	709.17	691.98	<0.001
	20	1085.87	1072.59	22.25	24.56	-0.002	-0.35	-0.46	-0.07	1083.23	1070.86	<0.001
Gamma-log	0.10	416.01	411.01	21.74	22.02	-0.38	-0.36	-0.20	-0.21	412.44	411.33	<0.001
	0.25	238.23	231.27	28.83	29.07	0.01	0.02	-0.29	-0.66	236.45	230.76	<0.001
	0.50	167.64	141.00	40.69	41.18	0.44	0.50	0.75	0.38	167.21	138.98	<0.001
	0.75	100.59	74.04	66.84	68.00	1.21	0.66	3.12	-0.23	101.22	76.23	<0.001
		*l* × 100 = 95										
Normal-identity	5	769.39	771.61	3.65	3.87	-0.05	0.31	0.62	-0.31	768.22	770.51	<0.001
	10	784.90	788.64	7.91	8.55	0.08	0.44	0.20	0.12	783.50	788.0	<0.001
	15	799.72	805.98	11.29	12.97	-0.09	0.13	0.18	0.39	799.31	805.51	<0.001
	20	814.9	824.70	13.81	15.19	0.03	0.32	0.26	-0.49	812.96	824.31	<0.001
Gamma-identity	0.1	915.64	936.89	35.23	36.23	0.21	0.26	-0.17	-0.02	915.98	935.69	<0.001
	0.25	1213.14	1267.29	105.12	108.52	0.76	0.07	2.15	-0.25	1204.05	1257.17	<0.001
	0.50	1779.15	1933.00	223.41	234.89	0.51	0.32	0.24	-0.25	1715.71	1911.22	<0.001
	0.75	2179.78	2437.31	287.23	296.12	1.11	0.97	0.83	0.86	2176.32	2435.23	<0.001
Normal-log	5	470.21	480.24	2.87	3.12	-0.49	0.44	0.42	0.07	469.25	481.26	<0.001
	10	615.24	631.02	12.23	15.28	0.27	0.19	0.38	0.02	614.28	630.23	<0.001
	15	785.19	805.65	18.23	20.21	-0.11	0.16	-0.31	-0.24	784.12	807.34	<0.001
	20	1190.73	1223.51	25.22	26.33	0.11	0.22	-0.31	-0.41	1192.61	1225.25	<0.001
Gamma-log	0.10	574.62	594.03	27.14	27.17	-0.01	0.17	-0.14	-0.33	572.33	592.31	<0.001
	0.25	842.12	896.99	36.78	36.99	0.09	-0.25	-0.21	0.33	844.88	898.92	<0.001
	0.50	874.45	976.23	57.51	58.94	0.22	0.12	0.44	-0.29	879.97	978.79	<0.001
	0.75	1357.37	1697.97	127.030	131.23	1.23	0.67	3.21	1.23	1342.45	1701.12	<0.001

Following [46], to evaluate the performance of our SP model, we compare the TCs obtained in 10 simulated instances of the indicated distribution, link function, *σ* and percentiles with respect to a deterministic solution of out-of-sample scenarios generated from same parameters and distributions. The results of average increase of TCs (Δ and MAD) are reported in Table 7. Note that, in all cases, the performance of the SP models in terms of Δ and MAD, when modeling the DPUT via GLM or GARMA estructures, are greater than when considering a deterministic scenario, which does not consider uncertainty. In all tested instances, savings are obtained in TC, since Δ = MAD. In general, the performance decreases as *σ* increases, independent of the distribution and link function considered. In general, for the 95-th percentile of DPUT, performance in terms of Δ and MAD is slightly better than when considering the 5-th percentile, while the results are similar when comparing the link function. This performance is better in the normal distribution than in the gamma distribution and with the GARMA model, independent of the link function considered.

**Table 7:** Evaluation of out-of-sample scenarios for the indicated distribution, link, *σ* and percentile with simulated data and the mentioned performance indicator.

Distribution-link function	*σ*	Δ		MAD	
		GARMA	GLM	GARMA	GLM
		*l* × 100 = 5			
Normal-identity	5	38.76	38.63	38.76	38.63
	10	38.65	38.41	38.65	38.41
	15	37.96	37.64	37.96	37.64
	20	37.41	37.08	37.41	37.08
Gamma-identity	0.10	33.97	33.22	33.97	33.22
	0.25	28.25	25.44	28.25	25.44
	0.50	20.53	13.28	20.53	13.28
	0.75	5.46	3.24	5.46	3.24
Normal-log	5	39.30	39.07	39.3	39.07
	10	38.78	38.23	38.78	38.23
	15	38.15	38.01	38.15	38.01
	20	37.92	37.06	37.92	37.06
Gamma-log	0.10	34.12	33.49	34.12	33.49
	0.25	29.12	26.76	29.12	26.76
	0.50	20.98	15.12	20.98	15.12
	0.75	6.23	4.21	6.23	4.21
		*l* × 100 = 95			
Normal-identity	5	39.44	38.95	39.44	38.95
	10	38.73	38.52	38.73	38.52
	15	38.15	37.89	38.15	37.89
	20	37.71	37.46	37.71	37.46
Gamma-identity	0.1	34.00	33.24	34.00	33.24
	0.25	28.43	25.88	28.43	25.88
	0.50	21.03	14.01	21.03	14.01
	0.75	6.01	3.79	6.01	3.79
Normal-log	5	39.32	39.11	39.32	39.11
	10	38.98	38.55	38.98	38.55
	15	38.34	38.23	38.34	38.23
	20	38.12	37.45	38.12	37.45
Gamma-log	0.10	34.17	33.52	34.17	33.52
	0.25	29.15	26.96	29.15	26.96
	0.50	21.03	15.97	21.03	15.97
	0.75	6.83	4.65	6.83	4.65

### 5.2 Empirical illustration

First, we describe the problem and data set. Then, we provide an exploratory data analysis, verify the assumptions of the DPUT distribution and estimate the inventory model parameters.

The drug supply in pharmacy units of Chilean primary healthcare centers is channeled through their central warehouse, which is provided by an external supplier. The warehouse acts as an intermediary between suppliers and output units, whereas these output units receive the demand for drugs, including its own pharmacy, which dispenses prescriptions to patients. The central warehouse needs to store, conserve and distribute such drugs, delivering the products monthly to all output units by using aggregated demand requirements for each of them in the same periods of the decision stages.

To validate the proposed methodology, we use real-world monthly demand (*Y*) data of a pharmaceutical product correlated with the known demand of other pharmaceutical product (*X*). The statistical correlation between demands of these two products is often detected, because frequently more than one drug is used to treat the same disease. Thus, in the GARMA model, the DPUT of a product can co-vary as a predictor of the DPUT of other product. These products are shipped from the warehouse and delivered to a healthcare center, located at the city of Concon, Chile. This case study is based on unpublished real-world data collected during 48 months in 2012-2015 (from 01-January 1 to 31-December) for evaluating supply policies. The data set is displayed in Table 8.

**Table 8:** Data set of monthly DPUTs of two pharmaceutical products.

Month	1	2	3	4	5	6	7	8	9	10	11	12	13	14	15	16
*Y*	3174	3304	3053	3119	3245	3331	3246	2565	2625	2790	2668	2773	3075	3097	2974	2841
*X*	250	245	250	240	246	251	244	247	248	255	251	245	244	245	245	248
Month	17	18	19	20	21	22	23	24	25	26	27	28	29	30	31	32
*Y*	2148	2883	2857	2895	2632	2593	3046	2749	2980	2506	2511	2764	2425	2852	2566	2155
*X*	251	246	245	253	245	244	251	250	243	248	245	245	250	246	249	250
Month	33	34	35	36	37	38	39	40	41	42	43	44	45	46	47	48
*Y*	2445	2421	2661	2291	2603	2487	2425	2448	2236	2223	2352	2525	2699	2613	2114	2551
*X*	246	247	250	250	244	242	248	249	252	248	244	246	249	245	253	247

**Fig 1:**
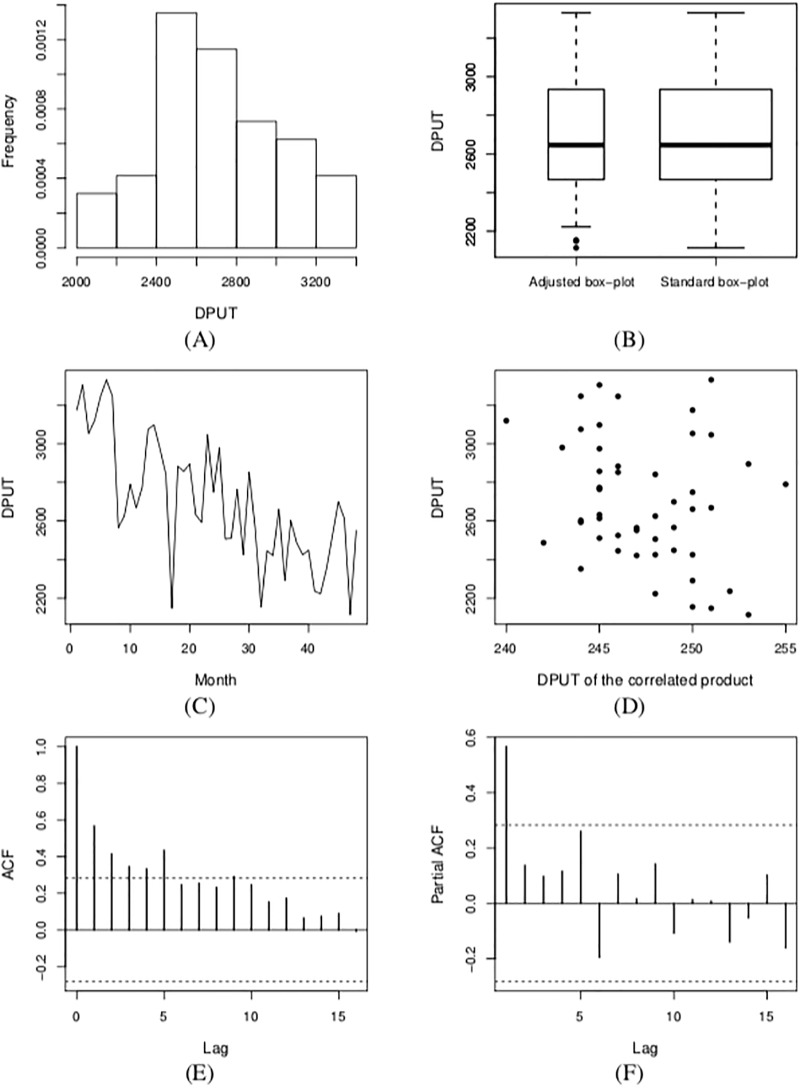
Histogram (A), box plots (B) and index-plot (C) of monthly DPUT; scatter-plot between DPUT and DPUT of the correlated pharmaceutical product (D); ACF (E) and PACF (F) plots of DPUT.

We perform an exploratory data analysis based on Table 9 and Fig 1. Table 9 reports the sample values of DPUT corresponding to: the mean ($\bar{y}$), minimum (*y*_(1)_), maximum (*y*_(48)_), inter quartile range (IRQ), 1st quartile (*y*_(12)_), median (*y*_(24)_), 3rd quartile (*y*_(36)_), SD, CS, CK and coefficient of variation ($\text{CV}=(\text{SD}/\bar{y})\times 100\%$). Fig 1 shows the histogram, standard box plot, box plot adjusted for asymmetric data [50] of DPUT, index-plot of monthly DPUT, scatter-plot between DPUT and DPUT of other correlated products, and ACF/PACF plots of DPUT data. We confirm the stationary nature of the time series with *p* = 2 using the Dickey-Fuller augmented test (results omitted here). From Table 9 and Fig 1(A) and 1(B), note that the median and mean are similar, the CS and CK are close to zero and three, respectively, indicating normality in the DPUT data, which is supported by the histogram and box plots. Fig 1(C) shows a decreasing trend in the DPUT for the studied period. From the scatter-plot presented in Fig 1(D), it is not easy to identity a suitable link function. Then, we use the logarithmic and identity link functions and compare them. Fig 1(E) and 1(F) provides evidence about seasonality, which can be modeled by an autoregressive component, whereas the component of moving average is not identified by these plots. In summary, from this exploratory data analysis, we assume a GARMA(*p*, *q*) model, with *p* = {0, 1, 2} and *q* = {0, 1} using a normal distribution for the DPUT and considering a covariate with different link functions for the mean response. To select the best GARMA model, we employ AIC, BIC and deviance, which are reported in Table 10. Note that, as mentioned in Subsection 4.2, GARMA(0, 0) model ≡ GLM.

**Table 9:** Descriptive statistics of monthly DPUT for the pharmaceutical product.

*n*	*y*_(1)_	*y*_(12)_	*y*_(24)_	$\bar{y}$	*y*_(36)_	*y*_(48)_	SD	IQR	CV	CS	CK
48	2115.0	2477.3	2647.0	2699.1	2915.5	3331.0	322.8	438.3	12.0%	0.2	2.2

**Table 10:** Criterion and deviance for different GARMA models with DPUT data of the pharmaceutical product.

Information criterion	GARMA(0, 0)		GARMA(1, 0)		GARMA(0, 1)		GARMA(1, 1)		GARMA(2, 0)		GARMA(2, 1)	
	Identity	Log	Identity	Log	Identity	Log	Identity	Log	Identity	Log	Identity	Log
AIC	692.621	692.55	666.94	658.45	659.27	659.29	654.82	651.06	646.73	869.17	646.78	869.17
BIC	698.235	698.17	674.43	665.94	668.62	668.64	654.17	660.42	657.96	880.40	658.00	880.41
Deviance	686.621	686.55	658.94	650.45	649.27	649.29	634.82	641.06	634.73	857.17	634.83	857.17

From Table 10, note that the smallest AIC, BIC and deviance correspond to the GARMA(2, 0) model, which corroborated our conjecture from the exploratory data analysis. Therefore, to model the DPUT, we propose the GARMA model given by

$$E\left(Y_{j}\right)=\mu_{j}=\beta_{0}+\beta_{1}x_{j}+\phi_{1}(y_{j-1}-\beta_{0}-\beta_{1}x_{j-1})+\phi_{2}(y_{j-2}-\beta_{0}-\beta_{1}x_{j-2}),$$ (18)

where *β*_0_ and *β*_1_ are the regression coefficients, *x*_*j*_ is the value of the covariate *X*, and *φ*_1_, *φ*_2_ are the autoregressive coefficients. We fit the GARMA model by using the garmaFit command. The maximum likelihood estimates of the model parameters given in Eq (18), with approximate estimated standard errors in parenthesis, are: ${\hat{\beta }}_{0}=6971.76\left(2469.09\right)$, ${\hat{\beta }}_{1}=-17.49\left(9.95\right)$, ${\hat{\phi }}_{1}=0.45\left(0.10\right)$, ${\hat{\phi }}_{2}=0.14\left(0.14\right)$ and ${\left(\hat{\text{Var}}\left(Y_{t}|H_{t}\right)\right)}^{1/2}={\left(\hat{\phi }\left(\mu_{t}\right)\right)}^{1/2}=240.15\left(25.03\right)$. All coefficients are significant at 10%. This conducts to the predictive model expressed as

$${\hat{\mu }}_{j+1}=6971.76-17.49x_{j+1}+0.45\left({\hat{y}}_{j}-6971.76+17.49x_{j}\right)+0.14\left({\hat{y}}_{j-1}-6971.76+17.49x_{j-1}\right).$$

**Fig 2:**
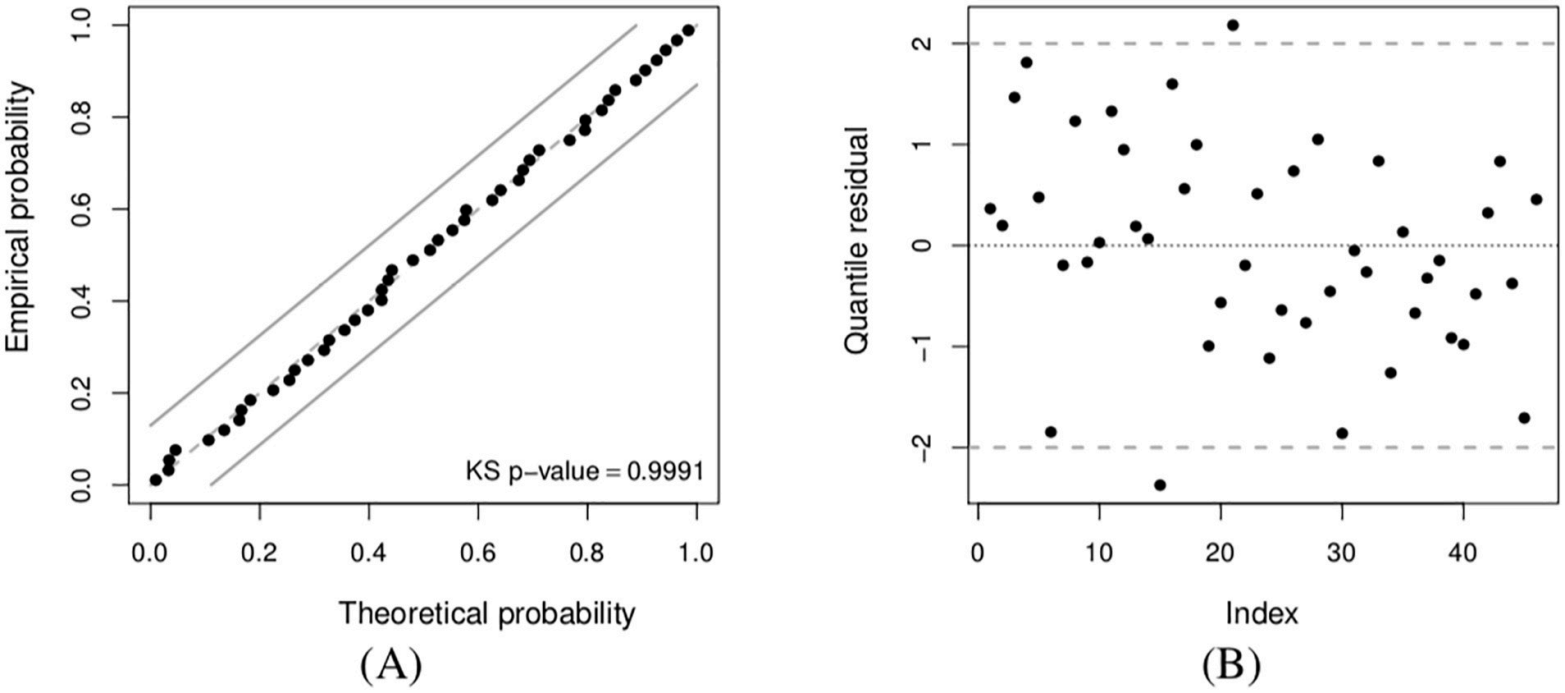
PP plot with 95% acceptance bands (A) and index-plot of the quantile residual (B) with DPUT data.

To confirm the correct fit of the proposed GARMA(2, 0) model in Eq (18), we use the quantile residual (*r*_*j*_) defined in Eq (9) for DPUT data. In addition, we employ a theoretical probability versus empirical probability (PP) plot to do this evaluation. Note that the PP plot can be linked to the Kolmogorov-Smirnov (KS) test by means of which acceptance bands may be constructed inside of this plot. Fig 2(A) sketches a PP plot with 95% acceptance bands to verify the distributional assumption of the model given in Eq (18). Observe that the KS *p*-value is 0.9991, which strongly supports the normality assumption of the quantile residuals obtained from the GARMA(2, 0) model. Fig 2(B) displays an index-plot of this residual, from which no unusual features, such as neither outliers nor heterogeneity, are detected. Therefore, from Fig 2, the assumption that the response follows a normal distribution seems to be quite suitable.

Once the statistical model has been identified and estimated based on the available DPUT data, the two-stage SP must be conducted to obtain the values of two sets of decision variables. In its first stage, this indicates both whether the binary variable *Z*_*t*_ takes the value one (a purchase must be carried out) in the period *t* or the value zero for *Z*_*t*_, and if it corresponds, the ELS *Q*_*t*_ to be purchased in this period *t*. In its second stage, once the DPUT fitted values are obtained, these values are organized in a scenario representation of two nodes for each period *t* = 1, 2, 3. To demonstrate how accurate the ELS inventory model is, depending on GLM or the GARMA(2, 0) model, we generate scenarios of 5-th and 95-th percentiles of the DPUTs ($y_{t_{5}}^{\omega },y_{t_{95}}^{\omega }$) and their probabilities ($p_{t_{5}}^{\omega },p_{t_{95}}^{\omega }$) by using sequential simulation according to [37]. The parameters employed for this simulation are: (i) holding cost of each period (*h*_*t*_) of 0.0035 USD$/(month per unit); (ii) unitary purchase cost in each period (*u*_*t*_) of 0.6 USD$/unit; (iii) shortage cost in each period of 0.33 USD$/(shortage unit); and (iv) order cost in each period of 0.86 USD$/order. We compare outcomes of SP for the ELS with inventory shortage in two stages, with and without temporal structure, which is reported in Table 11. Note that the results for E(TC) and quantities *Q*_*t*_ to order in each period are smaller and more accurate when the DPUT is described by a GARMA(2, 0) model than by GLM. This is because the values of $y_{t}^{\omega }$ obtained with a GARMA model are smaller and have higher accuracy than using GLM.

**Table 11:** Effect of scenarios of DPUT percentiles using the indicated model on SP elements in two stages with DPUT data of the pharmaceutical product.

Percentile	Model	$\tilde{E}\left(\text{TC}\right)$	${\tilde{Q}}_{1}$	${\tilde{Q}}_{2}$	${\tilde{Q}}_{3}$
5	GARMA(2, 0)	3846.44	2043	2098	2054
	GLM	4118.61	2162	2276	2223
95	GARMA(2, 0)	5271.31	2834	2854	2823
	GLM	5939.97	3187	3280	3224

To evaluate the performance of SP with respect to deterministic out-of-sample scenarios, we divide our data set into two parts. We use 24 of 36 observations to estimate the parameters of GARMA and GLM structures. Then, we make predictions for 12 periods of future decision stages, as expressed previously, and solve the SP as indicated in Algorithm 2. The remaining 12 observations of the sample are used to compare the behavior of the stochastic solutions with respect to the deterministic solutions. The results of average increase of TCs (Δ and MAD) are reported in Table 12. These results are consistent with those found in our simulation study, since they indicate that the performance of SP obtained by modeling demand with GARMA model are better than when the uncertainty of this variable is not considered.

**Table 12:** Evaluation of out-of-sample scenarios for the case study according to the mentioned performance indicator.

Performance indicator	Percentile	GARMA	GLM
Δ	5	36.79	36.59
MAD	5	36.79	36.59
Δ	95	37.60	37.49
MAD	95	37.60	37.49

## 6 Conclusions, limitations and future research

This research proposed a methodology to solve a probabilistic ELS problem under time-dependent demand. A two-stage SP approach and a GARMA model for generation of scenarios were considered. Our results reported that scenarios with temporal dependence provide more accurate estimates for lot-sizing and smaller amounts of stored and shortage items in the different periods of the decision stages, when compared to time-independent DPUT, represented by GLM. The methodology proposed in this work showed an interesting approach to achieve savings in inventory total costs, assuming the variability of DPUT scenarios linked to time-series. This approach improved the existing results, preventing both unnecessary stock-outs and inventory holding. The approach offered the advantage of considering a more realistic and accurate situation of the DPUT. The proposed ELS policy is useful in organizations that have a single supplier to meet their requirements and that are generally characterized by high bureaucracy in their administrative systems. This is the case of public hospitals, where such a behavior in pharmaceutical product DPUT is frequent, and therefore, the supply system can be facilitated [51]. GARMA models give the possibility of achieving an efficient characterization of the mean, as well as the adjustment of other parameters, which may be used in probabilistic ELS problems. Although we employ a normal distribution in this paper, the proposed methodology is valid for any distribution that may be parameterized with respect to the conditional mean of a time-series model, considering linear and non-linear link functions for describing the covariates. On the one hand, this parametrization provides a basis for generating useful scenarios in SP. On the other hand, forecasting to future values based on GARMA models also gives the possibility of evaluating the quality of the prediction.

Note that the out-of-sample scenarios used in the simulation correspond to values based on the same parameters and statistical distribution under consideration. However, the out-of-sample scenarios used in the empirical illustration correspond to estimated values based on the real data under analysis. In both simulation and empirical studies, our results of SP confirm an important improvement in the function of total costs. These differences are slightly higher when considering the modeling of the demand through a GARMA model than when using a GLM.

Some limitations of the present research are that we do not consider: (i) random lead times nor restrictions on the fill-rate or service levels; and (ii) multiple products. When there is access to data related to time-dependent demand for more than a single product, the methodology proposed in this study could be extended to multiple products. These limitations generate opportunities for new approaches that take into account such aspects in two-stage SP. Furthermore, as future research, from the point of view of inventory models, we can employ approaches which study restocking cycles with scenarios of random lead time. Also, the use of chance constrains to consider fill-rate or service level may be assumed. From the point of view of statistics, it is possible to improve the statistical modeling if distributions different than the normal model are considered. Furthermore, given that in many products the DPUT in each cycle can be zero, a model employing mixture probability distributions for continuous and/or discrete data, called zero-inflated, may be considered [48]. Also, since outliers are known to be harmful for the estimates of statistical model parameters, diagnostic tools can be considered for the approach proposed in the present work. Even multivariate versions of the statistical models may be assumed to improve the accuracy of the proposed approach [38, 52–54].
